# Obesity and microbiota: an example of an intricate relationship

**DOI:** 10.1186/s12263-017-0566-2

**Published:** 2017-06-15

**Authors:** Sabrina Duranti, Chiara Ferrario, Douwe van Sinderen, Marco Ventura, Francesca Turroni

**Affiliations:** 1 Laboratory of Probiogenomics, Department of Chemistry, Life Sciences and Environmental Sustainability, University of Parma, Parco Area delle Scienze 11/a, 43124 Parma, Italy; 2 APC Microbiome Institute and School of Microbiology, National University of Ireland, Cork, Ireland

**Keywords:** Microbiota, Obesity, Diet, Probiotics

## Abstract

It is widely accepted that metabolic disorders, such as obesity, are closely linked to lifestyle and diet. Recently, the central role played by the intestinal microbiota in human metabolism and in progression of metabolic disorders has become evident. In this context, animal studies and human trials have demonstrated that alterations of the intestinal microbiota towards enhanced energy harvest is a characteristic of the obese phenotype. Many publications, involving both animal studies and clinical trials, have reported on the successful exploitation of probiotics and prebiotics to treat obesity. However, the molecular mechanisms underlying these observed anti-obesity effects of probiotics and prebiotic therapies are still obscure. The aim of this mini-review is to discuss the intricate relationship of various factors, including diet, gut microbiota, and host genetics, that are believed to impact on the development of obesity, and to understand how modulation of the gut microbiota with dietary intervention may alleviate obesity-associated symptoms.

## Background

Diet and lifestyle have a crucial influence on the health status of humans, and it is widely accepted that various metabolic syndromes represent diet-induced diseases that account for one of the largest global health problems [1]. Nevertheless, the etiology of metabolic syndrome is multifactorial and apart from diet other variables such as host genetics and environmental factors are assumed to be involved. A growing list of publications have implicated the gut microbiota, i.e., the community of microorganisms residing in the gastrointestinal tract (GIT), as one of the major players involved in the development of certain metabolic syndromes [2]. In this context, there are several studies clearly showing that diet, and thus nutrient availability, modulates the composition and activity of the gut microbiota [3]. In particular, recent metagenomics-based studies have identified the gut microbiota as an environmental factor influencing whole-body metabolism by affecting not only energy balance but also immune and gut barrier functions [4, 5]. The human gut is considered a bioreactor with a huge diversity of bacterial taxa, predominantly belonging to the *Firmicutes* and *Bacteroidetes* phyla [6], and shaped by different environmental parameters.

Disruption of the state of homeostasis among members of the gut microbiota may cause imbalances among bacterial communities residing in the intestine, a situation that has been referred to as dysbiosis [7]. Dysbiosis is frequently associated with the development of a variety of diseases ranging from localized gastroenterological disorders to neurological, respiratory, metabolic, hepatic, and cardiovascular illnesses [8]. Obesity increases cardiovascular disease through different risk factors, i.e., elevated triglycerides, high low-density lipoprotein (LDL)-cholesterol, low high-density lipoprotein (HDL)-cholesterol, high blood pressure, and elevated blood glucose and insulin levels [2].

Diet, the clinical definition of which is the total food intake by an individual over a given time period, is believed to be linked to obesity with the gut microbiota also playing an important role [4]. Thus, the hypothesis that obesity can be controlled by modulating the gut microbiota may lead the way to effective therapeutic interventions [9, 10]. It is known that different environmental factors, including diet, influence the relative abundance of certain bacterial phyla in the gut and consequently their functional attributes, with an impact on host metabolism [11]. Gut microbiota of obese individuals exhibit reduced taxonomic diversity and consequent diminished metabolic capacity compared to the microbiota of lean individuals [12, 13]. Actually, an efficiently fermenting microbiota may promote an obesity status, whereas a low-efficient bacterial community may promote leanness due to reduced energy harvest from carbohydrate, as well as lipid fermentation [14].

The strong correlation between diet, gut microbiota, and obesity is gaining significant research interest [2, 15], especially in order to better understand the etiology of obesity and to generate novel prevention and treatment methods. The current review will focus on the importance of diet as the responsible factor for obesity, in particular through its effect on gut microbiota maturation during infancy. We will also discuss available approaches on how to exploit beneficial bacteria to influence the composition of the gut microbiota and thus to modulate energy harvest efficiency.

## Review

### The etiology of obesity

Obesity and overweight are defined as abnormal or excessive fat accumulation, resulting from an amount of ingested energy that is higher than the amount expended [16]. The World Health Organization (WHO) has defined an overweight individual as someone having a body mass index (BMI, i.e., the weight in kilograms divided by the height in meters squared) between 25.0 and 29.9 kg/m^2^, and an obese person as someone with a BMI greater or equal to 30.0 kg/m^2^ [16].

The worldwide prevalence of obesity has doubled during the last 30 years, and consequently, WHO has declared obesity as a current global epidemic. In 2014, about 1.9 billion adults were deemed to be overweight [17]. This number corresponds to about 39% of the world population older than 18 years. About 13% of wordwide adult population, corresponding to 600 million people, is considered obese [16]. Childhood obesity has increased exponentially in infants and young children (aged 0 to 5 years) during the past 25 years having increased from 32 million globally in 1990 to 42 million in 2013 [16, 18].

**Fig. 1:**
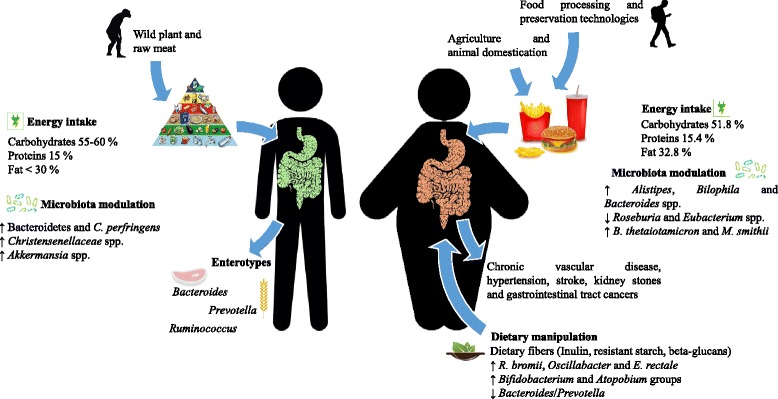
Schematic representation of the diet–microbiota–obesity correlations. Interactions between diet and gut microbiota in lean and obese subjects: nutrition, energy intake, and microbiota modulation are reported. For lean individuals, possible microbiota enterotypes are shown. For obese individuals, obesity-correlated diseases and possible dietary manipulations are illustrated

Obesity is considered to be a major risk factor for other metabolic complications, such as type 2 diabetes, insulin resistance, metabolic inflammation and non-alcoholic fatty liver disease, hypertension, and certain types of cancer [19, 20]. The incidence of type 2 diabetes, which is strictly correlated with BMI and age, is on the increase and has been reported to affect 422 million adults globally [21]. A high BMI is associated with risk of coronary heart disease and stroke [22]. High BMI and obesity are also associated with colorectal cancer in men and breast cancer in post-menopausal women [23] (Fig. 1).

The obesity pandemic is tightly linked to an increase in energy availability and sedentariness. The etiology of obesity is multifactorial and environmental, involving dietary, genetic, pathological, and lifestyle factors [24], though the individual contributions of these factors may not always be fully understood.

There is a strict correlation between diet, microbiota, and obesity [25]. As a clear example of this, carbohydrates are a vital source of energy for the human body, yet humans have very limited abilities to degrade and utilize dietary mono-, oligo-, or poly-saccharides [26]. Remarkably, various members of the gut microbiota, known as saccharolytic microorganisms, degrade these complex glycans thereby providing the host with a variety of metabolites, in particular short-chain fatty acids (SCFAs) which impact on glucose, cholesterol, and lipid metabolism [27, 28]. In addition, the ingestion of different types of food, such as different kinds of carbohydrates, may influence the gut microbiota composition [29].

Interestingly, the gut microbiota has been shown to regulate energy metabolism and fat storage, and is believed to be a driving force in the development of metabolic disorders associated with obesity [30].

### Obesity and diet

Obesity and metabolic syndrome in general are influenced by many physiological factors that are strongly associated with diet and lifestyle, in addition to genetic and environmental factors [31].

Before the development of agriculture and animal husbandry, human diet was necessarily limited to wild plants, berries, roots, and raw animal foods. With the domestication of plants and animals, and the processing of such foods, the original nutrient characteristics of these unprocessed foods have changed rapidly with advancing technology following the Industrial Revolution. Food products have profoundly changed during the last decades, thanks to various food processing and preservation technologies [32], modifications that have introduced food with higher (and more readily available) calories, yet with a lower nutritional value, as compared to fresh vegetables and fruits [33]. The ongoing westernization, urbanization, and mechanization processes that have happened in most countries around the world have resulted in a population with a sedentary lifestyle and a high fat content, high energy-dense diet [34]. As populations become more urbanized and with rising incomes, diets high in sugar, fat, and animal products replace the traditional diets that are rich in complex carbohydrates and fibers [35] (Fig. 1).

A general assumption is that a diet in which fat represents more than 30% of its total energy contributes to the development of obesity [36]. In a balanced diet, carbohydrates should represent the greatest portion of the energy intake (55–60%), with proteins contributing about 15% of the energy intake (and as mentioned above fat being no more than 30%) [37]. In a high fat diet (HFD), similar to a typical western USA diet, the percentage of total food energy derived from the three major macronutrients is as follows: carbohydrate (51.8%), fat (32.8%), and protein (15.4%), and with high levels of refined sugars, refined vegetable oils, and salt [38]. The health consequences of a HFD have been reported to be rather diverse (Fig. 1).

The modified fatty acid composition of a western diet, which is usually rich in saturated and *trans* fatty acids, increases the risk of chronic vascular disease by elevating (blood serum) concentrations of total and LDL-cholesterol [39].

An altered sodium-potassium ratio is caused not only by the high amount of salts in certain diets but also from the use of refined oil and sugars that are poor in potassium. Diets low in potassium and high in sodium may lead to a variety of chronic illnesses, including hypertension, stroke, kidney stones, and cancers of the gastrointestinal tract [40].

An important remedial role is played by dietary fibers that can reduce total and LDL-cholesterol concentrations by delaying gastric emptying, which may reduce appetite and thus help to control caloric intake [41]. Inulin, resistant starch, and beta-glucans are important dietary fibers, which have been well studied and shown to modulate gut microbiota [42]. Inulin has been reported to regulate gastrointestinal motility and appetite, reduce fat mass accumulation, and affect adipose tissue metabolism [43]. Ingestion of resistant starch and beta-glucans causes a satiating effect [44]. Sufficient fiber intake should amount to 25–30 g/day, while in a typical western diet this is about 15 g/day [37] (Fig. 1).

The change from a traditional diet to a western diet may alter the gut microbiota composition, thereby influencing various aspects of human health because of the strong correlation between diet and gut microbiota, as illustrated in the next section.

### Diet and microbiota

The diet represents one of the most important factors that determine gut microbiota composition [3]. The gut microbiota is now considered an organ, which regulates numerous physiological pathways and affects different host functions [45]. A substantial portion of gut physiology, including the modulation of gut motility, intestinal barrier homeostasis, nutrient absorption, and fat distribution, is believed to be influenced by the mutualistic relationship between intestinal microorganisms and their human host.

During the last decade, several studies have evaluated the influence of diet on the human gut microbiota composition and its consequent impact on metabolic functions [46, 47]. Interestingly, David et al. [3] showed that the human gut microbiota is rapidly modified by dietary changes. Different gut microbiota assemblies were shown to possess a varying capacity to produce particular metabolites, including SCFA, such as butyrate, phenolic acids, and branched-chain fatty acids [48]. Arumugam et al. [49] introduced a number of distinct gut microbial profiles, called enterotypes, which are currently believed to encompass three different microbial patterns dominated by *Prevotella*, *Bacteroides*, or *Ruminococcus* that are not restricted to a specific geographical origin [49]. However, enterotypes seem to be influenced by the diet followed by the host. In this context, individuals following a diet rich in protein and animal fat are associated with the *Bacteroides* enterotype, while *Prevotella* dominated individuals ingesting more carbohydrates [50] (Fig. 1). This indicates that only a limited number of well-balanced host-microbial symbiotic states exist that may respond differently to diet and drug intake. Nonetheless, despite the fact that the enterotype hypothesis is conceptually appealing, it has been subjected to a lot of scientific debate. In fact, to synopsize microbiota variation into three discrete clusters would imply that these enterotypes are relatively stable over time, a situation that is not true for healthy subjects where enterotypes are highly variable over time [51]. In addition, clustering methodologies can be sensitive to sampling bias and selection criteria prejudices [51].

Concerning the impact of different food constituents on the gut microbiota, the contribution of dietary fibers, such as resistant starch (RS) and inulin, as well as fat and proteins should be mentioned [15]. Notably, an RS-based diet was reported to provoke increased abundance of *Ruminococcus bromii*, as well as uncultured *Oscillabacter* and *Eubacteria rectale* [52]. In contrast, in the case of subjects following an inulin-based diet members belonging to the *Bifidobacterium* genus and *Atopobium* group were shown to undergo a significant increase in abundance, while members of the *Bacteroides*/*Prevotella* groups showed a reduction in relative numbers [53]. Furthermore, an HFD was shown to result in a modification of the gut microbiota composition and a stimulation of the bile acid secretion and increased fecal concentration of secondary bile acids [54]. In particular, this diet provoked an increase in the abundance of bile-tolerant microorganisms, such as *Alistipes*, *Bilophila*, and *Bacteroides* [3]. Finally, a high intake of protein and low carbohydrate intake was shown to result in a reduction in the abundance of *Roseburia* and *Eubacterium* [55]. Interestingly, a protein-rich diet decreased the production of butyrate as well as fiber-derived, antioxidant phenolic acids, and an increase in branched-chain fatty acids, which result from fermentation activities by gut bacteria [55]. Therefore, these combined data clearly demonstrate that the gut microbiota composition is a reflection of various dietary lifestyles.

### Links between obesity and host genetics

Recently, it has been demonstrated that host genetics has a direct influence on various metabolic syndromes, such as diabetes and obesity. Several studies performed in mice as well as in humans have revealed specific associations between host genotype and microbiota composition [56–59]. In this context, leptin, i.e., the so-called satiety hormone, plays different roles in human physiology by regulating appetite and body weight, and insulin secretion [60, 61]. Different studies have demonstrated that leptin genes (*lep*) and the corresponding leptin receptor (LEPR) exert a specific effect on the gut microbiota composition [62–65]. In detail, leptin-deficient *ob*/*ob* mice revealed an increased susceptibility to *Klebsiella pneumoniae* and *Streptococcus pneumoniae*, suggesting that leptin somehow protects against bacterial infection [66]. Furthermore, comparison between obese, leptin-deficient mice, and wild-type, lean mice, highlighted that the microbiota of obese mice was characterized by a lower abundance of *Bacteroidetes* [62]. In a rat model, the loss of the leptin receptor resulted in an increase in *Holomonas* spp. and *Sphingomonas* spp. and decreased levels of *Bifidobacterium* spp. [65].

Another gene that was shown to be involved in obesity as well as in cardiovascular disease is represented by *apo*A1 gene, which encodes for the Apoliprotein A1, and mutation of which increases the development of these diseases/metabolic disorders [67]. Notably, polymorphism of the *apo*A1 gene has been correlated with a different gut microbiota composition in mice, characterized by an enrichment of *Desulfovibrionaceae* and a depletion of members of the *Bifidobacteriaceae* family [68].

Another genetic trait, which has been shown to be involved in the development of obesity, is represented by the human phospholipase D1-encoding gene (*pld*1). The gene product of *pld*1 was shown to provoke glycerol phospholipid hydrolysis with the concomitant production of phosphatidic acid, which is an intracellular messenger implicated in several cellular processes, including obesity [69]. SNPs of *pld*1 gene were associated with abundance levels of genus *Akkermansia muciniphila* [70]. It has been proposed that a lower abundance of *A. muciniphila* may induce the development of obesity in mice [71]. Thus, the existence of correlations between a polymorphism of PLD1 and this bacterial taxon may represent an example of how host-associated genotype that is responsible for a specific gut microbiota profile, ultimately influencing the development of obesity.

### Microbiota and obesity

It is widely accepted that a high level of microbial complexity, i.e., high number of different microbial phylotypes present in healthy adult subjects, plays an important role in maintaining immune homeostasis [72]. In this context, comparison of gut microbiota differences between lean and obese individuals suggests that the microbiota of obese subjects is less complex [12], though this finding is still highly debated. An important functional sign that differentiates the microbiota of obese vs. lean individuals is represented by the fermentable ability exhibited by the members of the gut microbiota: this capability depends on the non-digestible dietary components introduced with the diet, which are ultimately crucial in SCFA generation by the gut microbiota [73]. These molecules affect host adiposity stimulating the hepatic de novo liponeogenesis, modulating the triglyceride storage, and consequently promoting energy storage [4]. Moreover, in healthy people, the produced SCFAs represent about 10% of the total energy obtained from the diet [74], and this energy is stored by the host body as fat [73]. SCFAs can be used as energy sources by the host but can also act as regulators of energy intake and energy metabolism. This notion is supported by the finding that germ-free mice, in which a gut microbiota is absent, gain less weight when fed with a high-fat diet compared to conventionally raised mice [75]. Moreover, obese, leptin-deficient (*ob*/*ob*) mice exhibit a gut microbiome enriched in genes involved in the recovery of energy from food [5]. Transplantation experiments of the gut microbiota from *ob*/*ob* mice and lean donors to germ-free mice provoked a significant increase in total body fat (from 27 to 47%) in mice colonized by the *ob*/*ob* mice microbiota. No significant increase in body fat was detected for mice colonized with the lean mice microbiota [5], demonstrating that the obesity-associated gut microbiota has an increased capacity to harvest energy from the diet.

Differences in the gut microbiota between lean and obese animals reveals that a microbiota, which is able to extract more energy from a given diet, is typified by a reduced presence of taxa belonging to the *Bacteroidetes* phylum and a proportional increase in members of the *Firmicutes* phylum [62]. The alteration of the microbiota equilibrium between *Firmicutes* and *Bacteroidetes*, with the increase of one phylum with respect to the other, was shown to be associated with a higher presence of enzymes for (complex) carbohydrate degradation and fermentation [5].

An interesting study aimed at investigating differences in the microbiota composition between obese and lean individuals in humans revealed significantly reduced levels of *Clostridium perfringens* and *Bacteroidetes* in obese compared to lean subjects [76]. Recently, a specific bacterial taxon was demonstrated to be associated with obesity, i.e., *Christensenellaceae* spp., and proposed as a novel microbial biomarker for obesity [77]. The member of this family was shown to reduce weight gain in mice and to modulate the gut microbiota composition [77]. In addition, other key members of the human gut microbiota, such as *Bacteroides thetaiotamicron* in association with *Methanobrevibacter smithii*, were shown to potentiate the process of adipose tissue accumulation [78]. Finally, *A. muciniphila* is associated with a healthier metabolic status, since it improved glucose homeostasis, blood lipid content, and body composition following a diet-imposed calorie restriction in humans [79]. However, these findings were simply associations since the causality between the presence of *A. muciniphila* and healthier metabolic status has not been established.

All these findings have corroborated the notion that obesity is correlated with a microbiota exhibiting an imbalanced *Firmicutes*/*Bacteroidetes* ratio, associated with an increase in *Actinobacteria* phylum and a decrease of *Verrucomicrobia* [12, 80].

There are ample publications which suggest correlations between body mass index and the presence of specific gut microorganisms [81–84]. In support of this, a recent clinical study involving 263 individuals, including 134 obese, 38 overweight, 76 lean, and 15 anorexic subjects, underlined the importance of a small number of microbial biomarkers that are linked to obesity, encompassing *Bacteroidetes*, *Firmicutes*, *M. smithii*, *Escherichia coli*, and various *Lactobacillus* species [85].

Only some of these microbial biomarkers such as *Bacteroidetes* or *M. smithii* have subsequently been confirmed in other studies [6, 12, 85, 86]. In this context, it should be mentioned that recent findings suggest the existence of a “dose-dependent” relationship between certain species of bacteria and archaea in the human gut, and BMI [85]. Specifically, a clear correlation exists between the number of *Lactobacillus reuteri* cells and obesity, where a higher abundancy of *L. reuteri* is associated with a higher BMI [85].

Recently, the fungal microbiota, i.e., mycobiome, has been characterized using Internal Transcribed Spacer (ITS)-based sequencing approach in obese and non-obese individuals [87]. Interestingly, this study showed that the mycobiome of obese subjects has an increased presence of the phylum *Ascomycota*, the class *Saccharomycetes*, and the families *Dipodascaceae* and *Saccharomycetaceae*, and an enhancement of fungi belonging to class *Tremellomycetes*, as compared with non-obese individuals. Specifically, *Mucor racemosus* and *Mucor fuscus* were the species more abundantly represented in non-obese individuals compared to obese counterparts indicating that the relative abundance of the *Mucor* genus increased after weight loss in obese subjects in a manner analogous to *Bacteroidetes* [87].

In order to overcome obesity, many dietary strategies have been put forward for effective weight reduction. However, most of these fail to maintain a long-term effect in reducing body weight. Intestinal microbiota alteration has been suggested to have a significant impact on this post-dieting period [88, 89]. Recently, a core microbiota that persists after successful dieting and weight regain has been found and this bacterial community contributes to enhanced metabolic derangement mediated by metabolite-induced effects on host metabolism [90]. It is proposed that this persisting microbiota may predispose the host to metabolic consequences in these repeated weight-gain cycles.

### Early microbe contact and the risk of obesity

The human intestine is considered sterile at birth [72], and the gut microbiota colonization process in infants commences during delivery and is enhanced by breastfeeding [91]. Recently, this hypothesis has been revised, even if it is largely debated, since it is proposed that an initial colonization process occurs during gestation [92]. Experimental evidence suggests that under normal gestational conditions bacteria from the maternal gut are transmitted into the mother’s blood stream and can ultimately either reside in the placenta or pass through the placenta and enter the amniotic fluid [93, 94]. The intestinal microbiota of neonates typically exhibits low diversity and a relative dominance of the phyla *Proteobacteria* and *Actinobacteria* [45, 72, 95, 96]. Several factors, such as gestational age, diet (e.g., breast milk vs. formula milk), sanitation, and antibiotic treatment are thought to influence the gut microbiota development and composition with the emergence and dominance of members of the *Firmicutes* and *Bacteroidetes* and reductions of other phyla, such as *Proteobacteria* and *Actinobacteria* [72]. Microbe-host interactions are considered crucial for the health of the host, and even at the first stages of life, such interactions are considered risk factors that impact on allergic diseases and development of obesity [72, 91, 97]. Another relevant and clear factor influencing the gut microbial development of the baby is the maternal weight-status (i.e., lean vs. obese). In this context, one study has highlighted that a child born from an obese mother possesses different levels of *Faecalibacterium* spp., *Oscillabacter* spp., *Blautia* spp., and *Eubacterium* spp. compared to a child born from a lean mother [98]. Moreover, it has been shown that the presence of a low concentration of *Bacteroides* spp. and a high level of *Lactobacillus* spp. in the newborn during the first 3 months of life may cause child obesity and overweight [99]. These data underscore the notion that the maternal microbiota is an important provider of microbes that colonize the infant gut and that this maternal microbiota transfer process impacts on the overall physiological conditions of the newborn [98, 99]. Thus, maternal obesity can be considered a predictor for child overweight [100]. Interestingly, another important factor that influences the gut microbiota composition of infants and affects the risk of obesity is human breast milk (HBM) [101]. HBM is a complex biological fluid that provides all necessary components, such as carbohydrates (lactose and oligosaccharides), fats, lipoglycans, proteins, enzymes, hormones, and microbes, for the development of the newborn [101]. Breast milk is not only considered crucial from a nutritional prospective but it also represents an important vehicle for vertical transmission of bacteria from mother to child [102–104]. It has been shown that the biodiversity of the infant gut microbiota is influenced not only by the maternal health status, mode of delivery, gestational age of the mother, and weight gain during pregnancy but also by maternal BMI [105, 106]. In particular, breast milk of obese mothers is characterized by a reduced microbiota diversity and distinct microbiota composition as compared to that from lean mothers, which was shown to contain a higher abundance of *Bifidobacterium* and lower counts of *Staphylococcus* [97]. Furthermore, Kalliomaki et al. [107] showed lower levels of *Bifidobacterium* spp. in infants that developed obesity during the first 7 years of their life compared to normal-weight children. This study proposed that the increased presence of *Bifidobacterium* spp. during the early stages of life may provide protection against overweight and obesity.

Another factor that may influence the development of pediatric obesity is exposure to antibiotics in early stages of life [108–110]. During infancy, which is the window of time before a stable microbial community has developed, the microbiota seems to have increased susceptibility to perturbations [111]. Assuming that intestinal microbiota can modulate host metabolism [5, 62, 112] it is, therefore, plausible that agents that specifically modulate the microbiota, such as antibiotics, can affect body weight. A greater nutrient absorption due to reduced bacterial populations, increasing production of microbiota-derived calories, reduction of microbial metabolites that inhibit absorption, and altered hepatic metabolic signaling and/or intestinal defenses are the proposed mechanisms [5, 108]. Of course, the variations in metabolic outcomes that are associated with antibiotic exposure seem to be largely dependent on the dose of antibiotics, timing of administration, and diet. Moreover, antibiotic use during pregnancy seems to have relevant consideration as discussed above, as infants acquire at least a part of their early life microbiota from their mothers [113, 114].

### Probiotic bacteria and prebiotic in prevention and treatment of obesity

Modulation of gut microbiota through the use of prebiotics and probiotics are claimed as possible strategies for the prevention of weight gain in obese-prone subjects and a non-invasive treatment for those individuals suffering from severe obesity.

Prebiotics are food components that confer health benefits to the host through the stimulation of growth and/or metabolism of beneficial bacteria including specific members of the gut microbiota [115]. Prebiotics generally include carbohydrates that are not accessible to the enzymes produced by the human body [e.g., fructo-oligosaccharides (FOS), galacto-oligosaccharides (GOS), gluco-oligosaccharides, xylo-oligosaccharides, inulin, resistant starch, arabinoxylan and arabinogalactan, lactulose, and raffinose], which means that they reach the distal sections of human GIT still intact where they constitute fermentable substrates for intestinal bacteria [116]. Prebiotics are considered as a nutritional tool to promote bacterial proliferation in the lower intestine, to induce modification of gut microbiota and, thus, to counteract fat mass accumulation and related metabolic disorders [117, 118].

In contrast, probiotics are health-promoting microorganisms, which are defined according to FAO/WHO as “live microorganisms that after ingestion confer health benefits to the host maintaining the gut microbiota correct equilibrium” [119], a definition recently updated as follows: “live microorganisms that, when administered in adequate amounts, confer a health benefit on the host” [120]. The health-promoting effects exerted by probiotic bacteria are mediated by the interaction with other gut-associated microorganisms and with the host [121]. Reported health benefits encompass modulation of the immune response, maintenance of the intestinal barrier, antagonism of pathogen adhesion to host tissue, and production of different metabolites such as vitamins, SCFAs, and molecules that act as neurotransmitters involved in gut–brain axis communication [122–124]. Currently, the large majority of commercially used probiotic bacteria belong to two genera, i.e., *Bifidobacterium* and *Lactobacillus*, both of which are typical inhabitants of the human intestine.

In a small number of cases, the role of particular *Lactobacillus* species on the modification of the body weight, both in animals and human beings, has been assessed [125]. However, the resulting data are rather contradictory and variable depending on the species as well as the strains involved. In this context, probiotic therapy based on *Lactobacillus rhamnosus* [126], *Lactobacillus delbrueckii* [127], and *L. reuteri* [128] was shown to result in weight increase perhaps because of their pro-inflammatory effects, while other *Lactobacillus* species, such as *Lactobacillus fermentum* [129] and *Lactobacillus gasseri* [130, 131] were associated with weight loss. Finally, certain species, such as *Lactobacillus sporogenes*, showed no significant effects on weight [125].

In a similar manner, species belonging to the *Bifidobacterium* genus, which represent microorganisms that are common inhabitants of mammalian GIT and which are associated with conferring beneficial effects on the host, were shown to have anti-obesity effects. For example, *Bifidobacterium pseudocatenulatum*, *Bifidobacterium longum*, and *Bifidobacterium adolescentis* have been shown to reduce body weight and blood serum levels (i.e., total cholesterol, HDL-cholesterol, LDL-cholesterol, triglyceride, glucose, leptin and lipase levels) [132, 133].

### Preclinical and clinical studies based on probiotic therapy

There are quite a number of studies reporting anti-obesity effects based on probiotic supplementation, which specifically act to mitigate lipogenesis, inflammation, and weight loss [4, 10, 134, 135]. Other trials suggest that the microbiota is involved in increased gut permeability for lipopolysaccharides, in lipogenesis, and in regulating fat storage and adiposity [4, 134, 136, 137].

An intriguing study has evaluated body weight effects as a result of supplementation of mice receiving an HFD for 12 weeks combined with a probiotic mixture encompassing both lactobacilli (*Lactobacillus paracasei* CNCM I-4270, *L. rhamnosus* I-3690) and bifidobacteria (*Bifidobacterium animalis* subsp. *lactis* I-2494). All strains significantly attenuated HFD-induced weight gain, improved glucose–insulin homeostasis, and reduced hepatic steatosis [10]. In addition, a significant reduction was observed of pro-inflammatory macrophage infiltration into adipose tissue, which is one of the causes of chronic adipose inflammation, insulin resistance, and other obesity-associated complications [10, 138].

Another preclinical study performed in diet-induced obese C57BL/6J mice treated with *Lactobacillus curvatus* HY7601 and *Lactobacillus plantarum* KY1032 for 10 weeks showed reduced body weight gain and fat accumulation, as well as lowered levels of plasma insulin, leptin, total-cholesterol, and liver toxicity biomarkers. All these data indicate that treatments with certain probiotic bacteria may counteract diet-induced obesity and modulate genes associated with metabolism and inflammation in the liver and adipose tissue [139].

*L. plantarum* strain HAC01 was shown to provoke similar effects of those described above, associated with a reduction of adipose tissue accumulation and a regulation of gene expression related to lipid metabolism in a diet-induced obesity murine model [140]. Various reports indicate that the benefits on body weight are mediated by metabolic effects, such as amelioration of lipid profile, improvements in insulin resistance, and control of glycemic values.

Various published studies have involved the probiotic *L. rhamnosus* GG strain [137, 141]. Apart from a physiological effect exerted by this strain in terms of reduction of weight gain in mice, it was observed that *L. rhamnosus* GG can protect animals from HFD-induced insulin resistance, as well as attenuate adiposity in the liver and mesenteric adipose tissue [141]. Notably, not only viable *L. rhamnosus* GG cells can elicit such an anti-obesity effect but also purified exopolysaccharides from *L. rhamnosus* GG cells can reduce adipogenesis and decrease fat pads and inflammation in mice through Toll-like receptor 2 expression in HFD-fed mice [137].

Another *L. rhamnosus* isolate, strain PB01, was also shown to exhibit anti-obesity activity. Specifically, strain PB01 was administered to diet-induced obese (DIO) mice and their normal-weight (NW) controls, resulting in weight reduction, which has been attributed to a protective effect of this strain on nociception circuits [142].

Similarly, the administration for 30 days of *L. paracasei* CNCM I-4034, *Bifidobacterium breve* CNCM I-4035, and *L. rhamnosus* CNCM I-4036 reduced hepatic steatosis in part by lowering serum lipopolysaccharide, and elicited an anti-inflammatory effect in obese rats [143].

A significant reduction of total body and visceral adipose tissue weight, together with improvement in insulin sensitivity was observed in Wistar rats following a short-term treatment with probiotic mixtures containing a concentrated biomass of 14 probiotic bacteria belonging to the genera *Bifidobacterium*, *Lactobacillus*, *Lactococcus*, and *Propionibacterium* [144]. Similarly, the individual administration of *Lactobacillus casei* IBS041, *Lactobacillus acidophilus* AD031, and two bifidobacterial strains, i.e., *Bifidobacterium bifidum* BGN4 and *B. longum* BORI, to HFD-fed mice for 8 weeks revealed potential anti-obesity effects of these strains. In fact, *B. longum* BORI was shown to significantly suppress murine weight gain and lower total cholesterol levels in the liver, while *L. acidophilus* and *B. bifidum* BGN4 were shown to significantly decrease triglyceride levels in the liver, showing a potential suppression of lipid deposition in this organ [145].

The effectiveness of probiotic therapy in the control/reduction of body weight was evaluated not only in animal experiments but also in human clinical trials. In this context, a group of women with excess body weight or obese phenotype (25< BMI <40) were enrolled for a randomized, double-blind trial to receive a probiotic mix based on *L. acidophilus* and *L. casei*, *Lactococcus lactis*, *B. bifidum*, and *Bifidobacterium lactis* for 8 weeks. Supplementation with a probiotic mix reduced abdominal fat and increased antioxidant enzyme activity as compared to dietary intervention alone [146].

Various studies have shown that bile acids act as signaling molecules in the host and thereby regulate energy, glucose, and lipid metabolism [147, 148]. Recently, it has been postulated that microbial metabolism of bile acids may also play a role in the regulation of host weight gain, particularly given that individual bile acids are regulators of host energy metabolism [135, 148, 149]. It is worth mentioning that numerous well-known probiotics exhibit bile salt hydrolase (BSH) activity [150] and this activity may partially account for their metabolic effects. It has been proposed that microbial BSH activity significantly alters both gastrointestinal and hepatic host functions [135, 149]. Using both germ-free and conventionally raised murine models, it was demonstrated that gastrointestinal expression of BSH results in local bile acid deconjugation with concomitant alterations in lipid and cholesterol metabolism, signaling functions, and weight gain. Moreover, it was shown that BSH mediates a microbe-host dialogue that functionally regulates host lipid metabolism confirming its role in metabolic syndrome [135]. Specifically, the expression of cloned BSH enzymes in the gastrointestinal tract of gnotobiotic or conventionally raised mice significantly altered plasma bile acid signatures and regulated transcription of key genes involved in lipid metabolism and gastrointestinal homeostasis. This high-level expression of BSH in conventionally raised mice caused a significant reduction in host weight gain, plasma cholesterol, and liver triglycerides, demonstrating the overall impact of elevated BSH activity on host physiology [135].

Apart from certain lactobacilli and bifidobacteria, other microorganisms, sometimes referred to as the next generation probiotics and belonging to *A. muciniphila* [71], *Pediococcus pentosaceus* [151], *Saccharomyces boulardii* [152], and *Bacteroides uniformis* [153] have been evaluated for their potential anti-obesity effects. Interestingly, the presence of *A. muciniphila*, a mucin-degrading bacterium that resides in the mucus layer of healthy individuals [154], was shown to be inversely correlated with body weight [155] and type 1 diabetes [156]. The abundance of *A. muciniphila* is significantly lower in obese mice, and a daily treatment with this bacterium to HFD-induced obese mice for 4 weeks normalized diet-induced metabolic endotoxemia and adiposity, reduced body weight, and improved body conformation, i.e., fat mass/lean mass ratio, without changes in food intake [71]. A specific analysis on the potential associations between specific microorganism and adipose tissue inflammation during obesity revealed that *A. muciniphila* abundance is inversely correlated with altered adipose tissue metabolism suggesting the existence of a link between the abundance of this species and adipose tissue homeostasis on the onset of obesity [157].

A clinical trial involving a dietary intervention of 49 overweight and obese adults displayed a significant association between *A. muciniphila* abundance and metabolic health. In fact, subjects with higher gene richness and *A. muciniphila* abundance exhibited the healthiest metabolic status, particularly in fasting plasma glucose, plasma triglycerides, and body fat distribution [79]. Recently, it has been demonstrated that treatment of mice with non-viable *A. muciniphila* cells, which had been killed by pasteurization, enhanced its capacity to reduce fat mass development, insulin resistance, and dyslipidemia [158]. This effect seems to be due to an interaction between Toll-like receptor 2 and a specific protein, which is not affected by the pasteurization process, and which is present in the outer membrane of *A. muciniphila* [158]. These findings suggest that non-viable *A. muciniphila* may be used as a therapeutic agent for the treatment of obesity and associated disorders.

A study involving *P. pentosaceus* LP28, administered to HFD-induced obese mice for 8 weeks, showed a reduced body weight gain and liver lipid contents (triglyceride and cholesterol) compared to a control group [151]. Recently, this strain was used in a clinical trial confirming the results obtained in the murine study. Furthermore, LP28 heat-killed cells display an anti-obesity effect that reduces BMI, body fat, and waist circumference, indicating that the LP28 strain represents a candidate for metabolic syndrome prevention/treatment [159].

As mentioned above, another probiotic strain tested for its potential anti-obesity feature is *B. uniformis* CECT 7771. This strain was administrated to obese C57BL/6 mice for 7 weeks, and the resulting effects included a reduced serum cholesterol, triglyceride, glucose, insulin, and leptin levels, improved oral tolerance to glucose, and a significant reduction in total body weight gain [153]. Recently, this strain was tested in a preclinical study to evaluate its safety following acute oral administration, revealing that no adverse effects were observed with regards to general health status or food intake [160].

Finally, the yeast *S. boulardii* was assayed for its anti-obesity properties. The administration of this strain to obese mice and type 2 diabetic mice for 4 weeks reduced body weight, fat mass, and hepatic steatosis, and caused a modification of the gut microbiota composition, which was characterized by a significant increment of *Bacteroidetes* and a reduction of the levels of *Firmicutes*, *Proteobacteria*, and *Tenericutes* phyla [152].

**Fig. 2:**
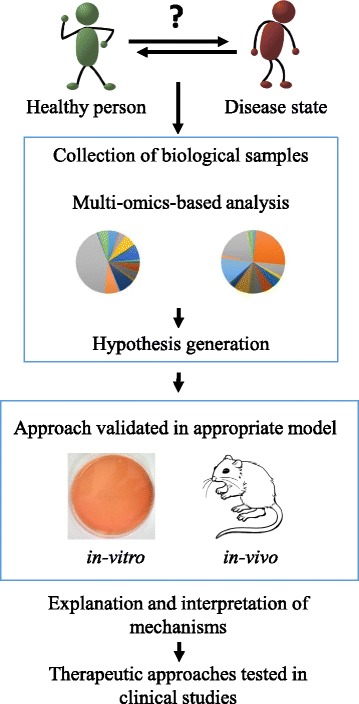
Strategies used to improve human health by gut microbiota modulation. Multi-omic approaches are employed to increase the understanding of how the microbiota may affect human metabolism. Such approaches will be crucial in order to dissect differences in the microbiota composition between healthy people and those who are affected by metabolic disorders. In a second step, different experimental in vitro and in vivo models are used to identify the underlying mechanisms responsible for the modulation the gut microbiota, which will be important to create the basis for human intervention trials and subsequent treatments

In summary, several preclinical and a small number of clinical studies involving different bacterial strains have been performed, showing that nearly all tested strains elicited varying anti-obese effects. However, in order to increase the understanding of such microbes and more specifically the various members of the gut microbiota that affect obesity in humans, multi-omic approaches involving key target tissues and a precise evaluation of microbiota composition should be combined for the generation of testable hypotheses. Such hypotheses will first have to be validated in animal models, followed by double blind, placebo-controlled interventions in humans (Fig. 2).

However, despite the increasing number of scientific reports on the anti-obesity activity of certain microbes, it is important to consider that the observed effects may vary dramatically from one individual to another as a consequence of the high inter-individual variability of the gut microbiota composition. In addition, the obtained results may also vary based on the tested microorganism, since the anti-obesity properties could be strain specific.

### Preclinical and clinical studies using prebiotics

Prebiotics are considered to exert anti-obesity activities through the selective modulation of specific microorganisms of the human gut microbiota. However, the underlying molecular mechanisms driving this response are far from being completely understood. Both animal and human clinical studies have investigated the potential anti-obesity features of various prebiotics, which associate the observed anti-obesity activity with alterations in hormone production, synthesis of SCFAs, and a decrease in bacterial lipopolysaccharides [155, 161–163]. Recently, the intervention with dietary inulin-type fructans (ITF) in 30 obese women for 3 months (16 g/day) selectively modified the gut microbiota composition, without provoking any significant change in host metabolism, and ultimately did not cause a significant effect on body weight [164]. In addition, treatment with ITF, but not the placebo, led to an increase in *Bifidobacterium* and *Faecalibacterium prausnitzii*. However, both bacteria negatively correlated with serum lipopolysaccharide levels. Similarly, a study enrolling 24 diabetic females that received (10 g/day) inulin or maltodextrin for 8 weeks (compared to a control group) revealed that inulin supplementation seems to modulate inflammation and metabolic endotoxemia in women with type 2 diabetes [165]. ITF consumption has also been reported to selectively modulate *Bifidobacterium* spp. and decreases fecal SCFA concentration in 15 obese women that received 16 g/day ITF for 3 months. These bacterial fermentation end products were shown to positively correlate with BMI suggesting that SCFA might be involved in body weight increase [166].

Inulin supplementation seems to have an impact on gastrointestinal hormones such as glucagon-like peptide-1 (GLP-1), peptide YY (PYY), ghrelin, and related peptide hormones both in animals [167–169] and in humans [43, 162, 170]. Emerging findings suggest that these enteroendocrine peptides are involved in the regulation of glucose homeostasis, energy balance, appetite sensations, and food intake [171, 172].

Prebiotic supplementation has been shown to influence not only appetite perception and fat mass storage but also host energy homeostasis. A satiating effect of resistant starch, supported by changes in neuronal activity in hypothalamic appetite regulation centers, has indeed been reported [44]. Similar results were obtained with the dietary supplementation of β-glucan that appears to have an effect on appetite regulation and an impact on energy intake [173].

On the other hand, a small number of studies have explored the effects of a prebiotic on liver diseases (i.e., inulin, raftilose, FOS, lactulose). Specifically, hepatic steatosis, which is characterized by abnormal lipid storage in liver, is closely linked to metabolic syndrome, as associated with obesity. In this context, it was shown that fermentable carbohydrates are involved in decreasing hepatic lipogenesis [174, 175].

Prebiotics seem to mediate microbial SCFA production, which has multiple roles in host homeostasis [176]. Specifically, prebiotics that are able to preferably stimulate the production of propionate and butyrate, as opposed to acetate, are of particular interest, since acetate constitutes a lipogenic and cholesterogenic substrate in the liver, while the production of propionate may decrease the hepatic lipogenic potential [177]. Nevertheless, this is depending not only on the prebiotic compounds but also on the presence of specific members of the gut microbiota promoting the conversion of these food ingredients into these particular SCFAs.

## Conclusions

The incidence of obesity and metabolic disorders has seen a dramatic increase among the human population in recent decades. Diet and lifestyle have an important impact on the development and evolution of obesity, though recent studies have also revealed the key role of the microbiota in the incidence and severity of this metabolic disorder. However, many details of the complex relationship that exists between microbiota, diet, and host remain to be unraveled. There is an urgent need for dietary interventions that provide unambiguous proof of the effectiveness of various dietary supplements (prebiotics and/or probiotics) with regards to modulation of the gut microbiota composition and/or metabolic activity to ultimately improve human health in the context of obesity. A better understanding of the impact of specific microbes on host physiology will be crucial in order to develop future therapeutic strategies to prevent and/or treat metabolic disorders including obesity. However, obesity is not only microbiota-driven thus a careful evaluation of all factors that are involved, including, but not limited to, host genetics, diet, and lifestyle, should be taken into account.

## Abbreviations

BMI: Body mass index
BSH: Bile salt hydrolase
DIO: Diet-induced obese
FOS: Fructo-oligosaccharides
GIT: Gastrointestinal tract
GOS: Galacto-oligosaccharides
HBM: Human breast milk
HDL: High density lipoprotein
HFD: High fat diet
ITF: Inulin-type fructans
ITS: Internal Transcribed Spacer
LDL: Low density lipoprotein
LEPR: Leptin receptor
NW: Normal weight
PYY: Peptide YY
RS: Resistant starch
SCFA: Short-chain fatty acids
WHO: World Health Organization
